# Supplementary material for: The association between non-high-density lipoprotein cholesterol to high-density lipoprotein cholesterol ratio (NHHR) and risk of prostate cancer: a retrospective study
Source: PeerJ. 2025 Mar 14;13:e19065. doi: 10.7717/peerj.19065 (PMC11913014; doi:10.7717/peerj.19065)
Supplement: Table S1 [file peerj-13-19065-s001.docx]

Table S1. Modified Poisson Regression

| **Variables** | **Unadjusted OR (95%CI)** | **P value** |  | **Model1 OR (95%CI)** | **P value** |
| --- | --- | --- | --- | --- | --- |
| NHHR | 0.96(0.93 ~ 0.99) | 0.033 |  | 0.95(0.91 ~ 0.99) | 0.008 |
| NHHR (Quantile) |  |  |  |  |  |
| *Q1* | Ref |  |  | Ref |  |
| *Q2* | 0.82 (0.70 ~ 0.95) | 0.007 |  | 0.84 (0.73~ 0.98) | 0.028 |
| *Q3* | 0.91 (0.79 ~ 0.95) | 0.200 |  | 0.89(0. 78~ 1.03) | 0.123 |
| *Q4* | 0.97 (0.85 ~ 1.11) | 0.685 |  | 0.92(0.79 ~ 1.07) | 0.259 |

Model1: Crude

Model2: Adjust: Leukocyte, Neutrophils, Erythrocyte, Triglyceride, LDL-C, Albumin, Globulin, AST, ALP, PSA.
