# Supplementary material for: The association between non-high-density lipoprotein cholesterol to high-density lipoprotein cholesterol ratio (NHHR) and risk of prostate cancer: a retrospective study
Source: PeerJ. 2025 Mar 14;13:e19065. doi: 10.7717/peerj.19065 (PMC11913014; doi:10.7717/peerj.19065)
Supplement: Table S2 [file peerj-13-19065-s002.docx]

Table S2 The diagnostic value of NHHR for prostate cancer

| AUC (95%CI) | Accuracy (95%CI) | Sensitivity (95%CI) | Specificity (95%CI) | PPV (95%CI) | NPV (95%CI) | Cut off |
| --- | --- | --- | --- | --- | --- | --- |
|  |  |  |  |  |  |  |
| 0.73 (0.70-0.76) | 0.69 (0.66-0.71) | 0.86 (0.84 - 0.89) | 0.51 (0.48 - 0.55) | 0.64 (0.61 - 0.67) | 0.79 (0.76 - 0.83) | 0.524 |
